# A comparison study on the behavior of human endometrial stem cell-derived osteoblast cells on PLGA/HA nanocomposite scaffolds fabricated by electrospinning and freeze-drying methods

**DOI:** 10.1186/s13018-018-0754-9

**Published:** 2018-03-27

**Authors:** Mojdeh Salehi Namini, Neda Bayat, Roxana Tajerian, Somayeh Ebrahimi-Barough, Mahmoud Azami, Shiva Irani, Saranaz Jangjoo, Sadegh Shirian, Jafar Ai

**Affiliations:** 1 Department of Biology, Science and Research Branch, Islamic Azad University, Tehran, Iran; 2 Brain and Spinal Cord Injury Research Center, Neuroscience Institute, Tehran University of Medical Sciences, Tehran, Iran; 3 Department of Tissue Engineering and Applied Cell Sciences, School of Advanced Technologies in Medicine, Tehran University of Medical Sciences, Tehran, Iran; 4 School of Medicine, Shiraz University of Medical Sciences, Shiraz, Iran; 5 Department of Pathology, School of Veterinary Medicine, Shahrekord University, Shahr-e Kord, Iran; 6 Shiraz Molecular Pathology Research Center, Dr Daneshbod Lab Pathology, Shiraz, Iran

**Keywords:** Bone tissue engineering, Endometrial stem cells, PLGA/HA scaffolds, Freeze-drying, Electrospinning

## Abstract

**Background:**

An engineered tissue structure is an artificial scaffold combined with cells and signaling factors. Among various polymers, the polylactide-co-glycolide/hydroxyapatite (PLGA/HA) has attracted much attention due to their optimal properties. The aim of this study was to study the behavior of human endometrial stem cell (hEnSC)-derived osteoblast cells cultured on PLGA/HA nanocomposite scaffolds.

**Methods:**

hEnSCs were isolated and exposed to osteogenic media for 21 days. Differentiated cells were cultured on PLGA/HA synthetic scaffolds. The PLGA/HA-based nanocomposite scaffolds were fabricated using either electrospinning or freeze-drying methods. Behavior of the cells was evaluated a week after seeding hEnSC-derived osteoblast-like cells on these scaffolds. Osteogenesis was investigated in terms of alkaline phosphatase activity, gene expression, immunocytochemistry (ICC), proliferation, and scanning electron microscopy (SEM). Moreover, scaffold properties, such as pore size and morphology of the cells, onto the scaffolds were evaluated using SEM. Furthermore, biocompatibility of these scaffolds was confirmed by 3-(4,5-dimethylthiazoyl-2-yl)-2,5-diphenyltetrazolium bromide (MTT) assay.

**Results:**

The matrix mineralization was proved by alizarin red staining, and the osteogenic media-treated cultures positively expressed osteocalcin and osteopontin markers. Moreover, qRT-PCR results confirmed the positive gene expression of osteopontin and osteonectin in the differentiated osteoblast-like cells. The results of behavior assessment of the cultured cells on electrospinning and freeze-dried scaffolds showed that the behavior of the cultured cells on the freeze-dried PLGA/HA scaffolds was significantly better than the electrospinning PLGA/HA scaffolds.

**Conclusion:**

It has been shown that the freeze-dried PLGA/HA nanocomposite scaffolds can appropriately support the attachment and proliferation of the differentiated osteoblast cells and are a suitable candidate for bone tissue engineering.

## Background

The musculoskeletal system is essential for its structural, protective, and support roles in the body as well as being a mineral source that facilitates movement [1]. Injuries to the musculoskeletal system are common, debilitating, and expensive to treat. Skeletal muscle injuries resulting in tissue loss are distinctively challenging in terms of surgical repair. Although the skeletal muscle is potentially regenerative, skeletal myofibers do not completely grow to fill the injured area, in case of losing a significant amount of tissue. If the defect does not exceed a certain volume, the healthy bone has the potency to regenerate [2]. However, in cases with extensive defects, bone graft biomaterials can be used for recovery of the defects and to facilitate bone formation in the defective regions [3]. Although, these traditional treatments have some limitations, such as disease transfer, histo-incompatibilities, limited autograft tissue supply, and insufficient mechanical support of implants or synthetic grafts. It has been shown that bone tissue engineering, as a new therapeutic strategy, can be used for bone regeneration [4–6]. Due to the drawbacks of the traditional therapeutic approaches, tissue engineering is being applied to look for new strategies to design an artificial biomaterial scaffold containing regenerating competent cells. Bone tissue engineering complex inclusive osteoconductive scaffolds, cells and osteogenic growth factors [7]. Among these three components, scaffolds play significant roles since they maintain the transplanted cells and lead their functions effectively [8–11]. For the artificial bone transplant, materials or the device must be non-toxic in interaction with body function [12, 13]. Hydroxyapatite (HA) (Ca10(PO_4_)6(OH)2) is known as the most biocompatible replacement biomaterial among the developed artificial bones, and it is a constituent of 70% of human [14, 15]. HA, as an alkaline calcium phosphate, is extremely bioactive and biocompatible due to its similarities to the bone tissue and mineral components of the tooth in the human body [16–20]. Furthermore, HA is the most widely used material for coating the hard tissue and metal implant due to being non-toxic and its ability to promote osteoinductivity [21, 22]. Poly(lactic-co-glycolic acid) (PLGA), as a copolymer of PGA and PLA, has significant properties, such as being mechanically strong and biodegradable as well as being Food and Drug Administration (FDA) approved. One of the most important advantages of PLGA is that its biodegradation can be controlled by altering the ratio of PLA and PGA; therefore, it has been widely used in the medical field [23]. Various fabrication methods have been evolved for constructing the scaffolds. Among these conventional methods, salt leaching, solvent casting, fiber bonding, phase separation processes, and membrane lamination approaches are currently used to fabricate scaffolds with irregular pore sizes and porosity [4]. Conventional methods, such as emulsion freeze-drying technique, were used for fabrication of highly porous PLGA scaffolds with an interconnected porous structure which is highly potential for bone tissue engineering [1]. Scaffolds with porosity greater than 90% and a pore size ranging from 20 to 200 μm can be fabricated using this method [3]. The pore size can be controlled by the freezing rate and pH; a faster freezing rate results in smaller pores [6]. Electrospinning is one of the widely used techniques for the preparation of nanofibrous materials with an ultrafine diameter (the diameter of the fibrous can range from few nanometers to several hundred nanometers or even micrometers), wide surface area per unit mass, and small interfibrous pore size [24]. Electrospinning has unique advantages over some other techniques that are used to fabricate scaffolds; for instance, the porous structures created using this method can potentially mimic the natural ECM of the biological tissues [25]. Electrospun nanofiber of biocompatible polymers is particularly used in drug delivery bioengineering, adhesion of biomacromolecules or cells, wound dressing, etc. [26]. It has recently been shown by several that mesenchymal stem cells (MSCs), embryonic stem cells (ES), and hematopoietic stem cells (HSC) can differentiate into osteoblast cells [27, 28]. Human endometrial stem cells (EnSCs) are an alternative for osteogenic differentiation due to their dynamic nature [29, 30]. The endometrial stem cells have shown to have a great multipotency potential. The human endometrium includes a few mesenchymal stem cells (MSCs) that can provide an easily accessible source of MSC. This study investigated the effect of the scaffold architecture on the adhesion, proliferation, and osteogenic differentiation of hEnSC-derived osteoblast cells cultured on PLGA/HA scaffolds which were fabricated using either freeze-drying or electrospinning techniques.

## Methods

### Differentiation of endometrial stem cells into osteoblast cells

#### Collection and culture of human EnSCs

Human EnSCs were isolated and purified from human endometrial tissue. Endometrial samples were collected from a reproductive-aged woman who was referred to the hospital for infertility treatment. The protocol for hEnSC extraction from the endometrium in this study has been previously reported [31, 32]. Flow cytometry analysis was performed to confirm the purity of the isolated stem cell cells. The list of antibodies that were used for flow cytometry in this study is as follows: CD105, CD90, CD31, CD34, and CD146. The identified hEnSCs were used for the experiments after passage 3 [33].

#### Osteogenic differentiation and alizarin red staining

hEnSCs were seeded at a concentration of 2 × 10^4^ cells/ml and then treated with osteogenic medium, as has previously been described by Shirian et al. [33].

#### Immunocytochemical analysis

The differentiated cells were fixed by being treated with 4% paraformaldehyde for 20 min, at 4 °C. Immunocytochemistry assay was performed on the osteoblast-like cells differentiated from hEnSCs cultured in osteogenic media for 21 days using specific antibodies targeting osteoblast cell markers, such as anti-osteopontin (mouse anti-human, Santa Cruz, USA) and anti-osteocalcin (mouse anti-human, Santa Cruz, USA), and were then incubated with secondary antibody (rabbit anti-mouse IgG-FITC, at a 1:700 dilution; Santa Cruz, USA) for 1 h, at 37 °C as previously described [33]. The stained cells were visualized using a fluorescence microscope (Olympus BX51, Japan).

#### Alkaline phosphatase production

The cells were seeded into the 24-well culture plates at a density of 1 × 10^3^ cells/cm^2^ in osteogenic culture media, for 21 days. The culture media were removed, and the cells were washed with PBS prior to being removed using a scraper and collected for experiments on days 1, 7, 14, and 21. The cells in one well of each plate were cultured without osteogenic media to be used as the negative control. Cells were centrifuged at 200*g* for 15 min and washed with PBS. Cell lysates were provided by vortexing the cells in 500-μl deionized water and 25 μl 1% Trito X-100 followed by sonification in order to obtain a homogenized lysate. The total protein content of the cells was specified using a commercially accessible kit (Micro/Macro BCA; Pierce Chemical Co., Rockford, IL). Moreover, the alkaline phosphatase (ALP) activity was measured, using a commercial kinetic kit (Pars Azmun, Iran), based on the transformation of p-nitrophenylphosphate to p-nitrophenol and phosphate at 37 °C and pH 9.8. The alterations in absorbance were monitored spectrophotometrically at 405 nm and temperature of 37 °C. ALP degrees was normalized to the total protein content of the cells at the end of the test [34, 35].

#### Quantitative real-time polymerase chain reaction

Real-time PCR was performed to detect the expression levels of osteoblast-specific genes, such as collagene type 1, Runx2, BGLAP, and IBSP at day 21 post-induction and 1 week after seeding the cells onto the mentioned scaffolds. The details of the primers used for RT-PCR are shown in Table 1. The differentiated hEnSCs to osteoblasts were isolated to extract the total RNA using TRIzol reagent (Gibco, USA). Cells were treated with DNase I, RNase-free kit (Takara, Bio, Inc., Shiga, Japan, 2270A) to remove genomic DNA. Complementary DNA was then synthesized using a Revert Aid First Strand cDNA Synthesis kit (Fermentas, USA, K1632). Relative gene expression analysis was evaluated with RT-PCR which was performed in 96-well optical reaction plates using a 7500 real-time PCR system (Applied Biosystems, USA) [34].

**Table 1:** Primer sequences used for QRT-PCR

Direction		Sequence of primer
COL1	Forward	ATGGCTGCACGAGTCACACC
COL1	Reverse	CAACGTCGAAGCCGAATTCC
BGLAP	Forward	GGTGCAGCCTTTGTGTCCAAG
BGLAP	Reverse	AACTCGTCACAGTCCGGATTGAG
IBSP	Forward	GATTTCCAGTTCAGGGCAGTAGTG
IBSP	Reverse	GTTTTCTCCTTCATTTGAAGTCTCCTC
RUNX2	Forward	ACTCTACCACCCCGCTGTCTTC
RUNX2	Reverse	AGTTCTGAAGCACCTGCCTGG
GAPDH	Forward	TCGCCAGCCGAGCCA
GAPDH	Reverse	CCTTGACGGTGCCATGGAAT

### Fabrication and characterization of nanocomposite scaffold

#### Scaffold fabrication

PLGA/HA scaffold was prepared using both electrospinning and freeze-drying methods. In order to fabricate PLGA/HA nanocomposite scaffolds using electrospinning method, PLGA (50:50, lactic acid to glycolic acid ratio, MW 48,000 w, Sigma-Aldrich) was dissolved in hexafluoroisopropanol (HFIP) using magnetic stirring for 2 h until the solution became clear. HA was added to this solution which was then stirred for 1 h at room temperature to obtain 10% (*w*/*v*) solution. The polymer solution was loaded into the 5-ml plastic syringe using a tip diameter of 22 gauges. The electrospinning processes were carried out using electrospinning vessel (Electroris®, Tehran, Iran). A high voltage of 15 kV was attached to the needle using a high voltage power supply. An aluminum foil was rolled on the Electroris grounded rotating drum as the collector and was placed at the distance of 15.0 cm from the needle tip. A syringe pump was applied to feed the polymer solution to the needle tip at a feeding rate of 1 ml/h. The nanofibers were collected over the aluminum foil. The electrospun fibrous were then dried under vacuum at room temperature, overnight. To fabricate freeze-dried PLGA/HA scaffolds using freeze-drying methods, PLGA was dissolved in HFIP using magnetic stirring at room temperature for 2 h. HA was then added to the solution and was stirred for 1 h to obtain 30% (*w*/*v*) solution. The prepared solution was then placed in the freezer (at − 80 °C) to be solidified. The solution was maintained in the freezer overnight. The solidified solution was placed into the freeze-drying vessel at − 10 °C. For complete separation of the water and solvent phases, the samples were freeze-dried for 10 h. The freeze-drying processes were carried out using freeze-drying equipment (Alpha 1-2 LD, Germany) [36].

### Scaffold characterization

#### Scanning electron microscopy

SEM was used to evaluate the microstructure and morphology of the nanocomposite scaffolds. Dry nanocomposite scaffolds were sputter-coated with a thin layer of gold (Au), and the morphology of the scaffolds was then evaluated using a scanning electron microscope (model Philips XL-30, Netherlands) at an accelerating voltage of 20 kV. The acquired images were used to evaluate the pore size of the nanocomposite scaffolds.

#### Cell attachment study using SEM

hEnSC-derived osteoblast cells were used to evaluate the in vitro cytocompatibility of the scaffolds. The cells were cultured in Dulbecco’s modified Eagle’s medium (DMEM)/F12 supplemented with 10% fetal bovine serum (FBS) and streptomycin/penicillin 100 U/ml (1%). To seed the cells onto the scaffolds, they were first trypsinized (0.05% trypsin/0.53 mM EDTA in 0.1 M PBS without calcium or magnesium) and then centrifuged prior to being resuspended in a complete culture medium. Finally, aliquots of 100 μl containing 50,000 cells were seeded on the top of each nanocomposite scaffold samples that were pre-soaked in the medium. Cells/scaffold constructs were kept in culture for 3 days at 37 °C in a humidified 5% CO2 incubator. To fix the cells/scaffold constructs, they were first pre-washed twice with PBS and then were soaked in 2.5% glutaraldehyde for 1 h at 37 °C. The samples were then washed with PBS and dehydrated in a series of consecutively increasing concentration of ethanol solutions (30, 50, 70, 80, 90, and 100%) at 37 °C for 15 min per concentration. Subsequently, the fixed samples were kept in *laminar* flow *hood* to be air-dried prior to being used for SEM observation [33].

### MTT assay

3-(4, 5-Dimethylthiazole-2-yl)-2, 5-diphenyl tetrazolium bromide (MTT) assay was used for the evaluation of cell viability by the measurement of the mitochondrial activity. MTT assay was performed on cultured cells onto the PLGA/HA scaffolds. The test was carried out using MTT (Sigma-Germany) on days 1, 3, 5, and 7 of culture as previously described [33, 34].

#### DAPI staining with scaffolds

The well of cells was fixed with 4% paraformaldehyde for 20 min at 4 °C and was then washed several times with PBS. For permeabilization, the cells were treated with 0.2% Triton-X 100 (Sigma-Aldrich) for 30 min. The non-specific binding sites were blocked with PBS/TWEEN. DAPI (4,6-diamidino-2-phenylindole, Sigma, USA) was then applied to the cells to stain the nuclei. The samples were then washed with PBS prior to being evaluated using a fluorescence microscope (Olympus BX51, Japan) [36].

### Statistical analysis

All data were analyzed by performing SPSS software. The results are presented as mean values ± standard deviation (SD). The data of proliferation and cytotoxicity assays were calculated by one sample *t* test. *P* values smaller than 0.05 were considered as statistically significant. We used random tests using REST 2009 software V2.0.13 for qRT-PCR to indicate statistical differences between groups.

## Results

### Identification of human EnSCs

Isolated hEnSCs were cultured in appropriate culture medium for 24 h. After about 10 days of being in culture, some heterogeneous adherent mesenchymal stem cells were obtained which were developed in numerous clusters. These cells were then used for subculture. After three passages, homogenous appearance of hEnSCs, elongated or spindle-like shapes, was observed. The results obtained from flow cytometry analysis which have been reported in our previous paper [32] showed that CD146+ (97%), CD105+ (79%), and CD90+ (80%) were extremely expressed in hEnSCs, and the expression of CD31− (0.02%) and CD34− (0.4%) were dramatically low in these cells.

### Matrix mineralization and differentiation analysis

#### Alizarin red staining

**Fig. 1:**
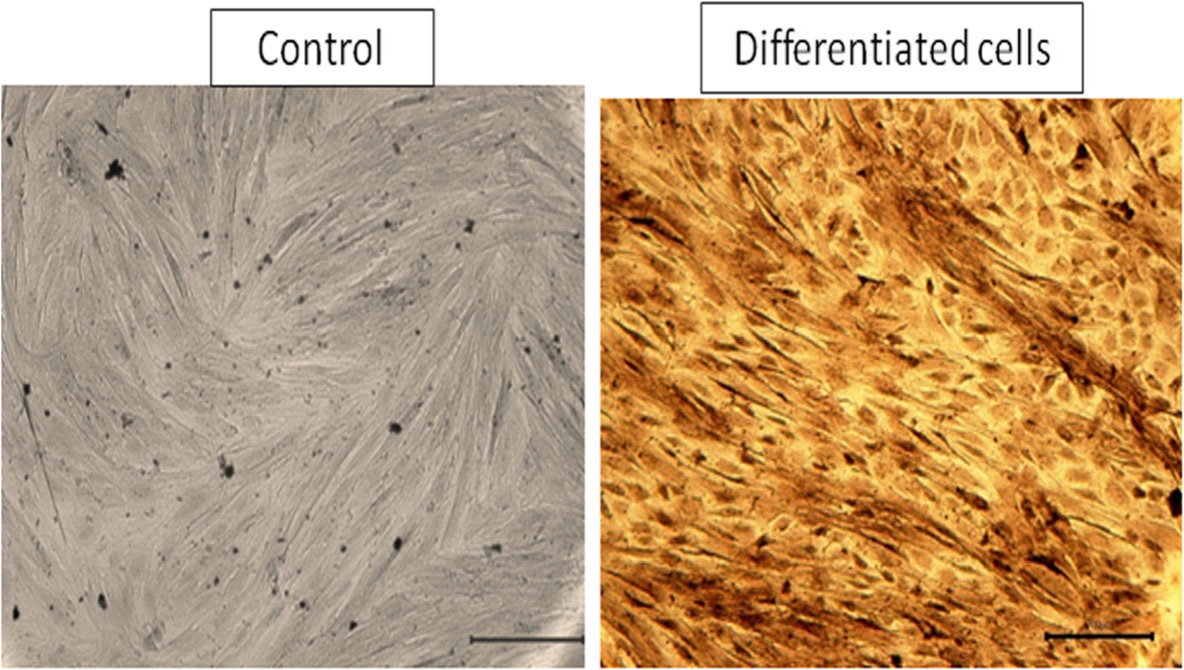
Alizarin red staining. Control (left panel). Endometrial stem cell-derived osteoblast-like cells (right panel)

The results of staining the cultured cells with alizarin red, after 21 days of being cultured in differentiation media, are presented in Fig. 1. Dark red stainings of calcium depositions were observed in the cells that were exposed to osteogenic media. No alizarin red staining was observed in the cells of the control groups. Based on the calcium deposition and calcium nodule formation, the treatment with osteogenic media resulted in osteogenic differentiation of hEnSCs into osteoblast-like cells after 21 days of culture.

### Immunocytochemical analysis

**Fig. 2:**
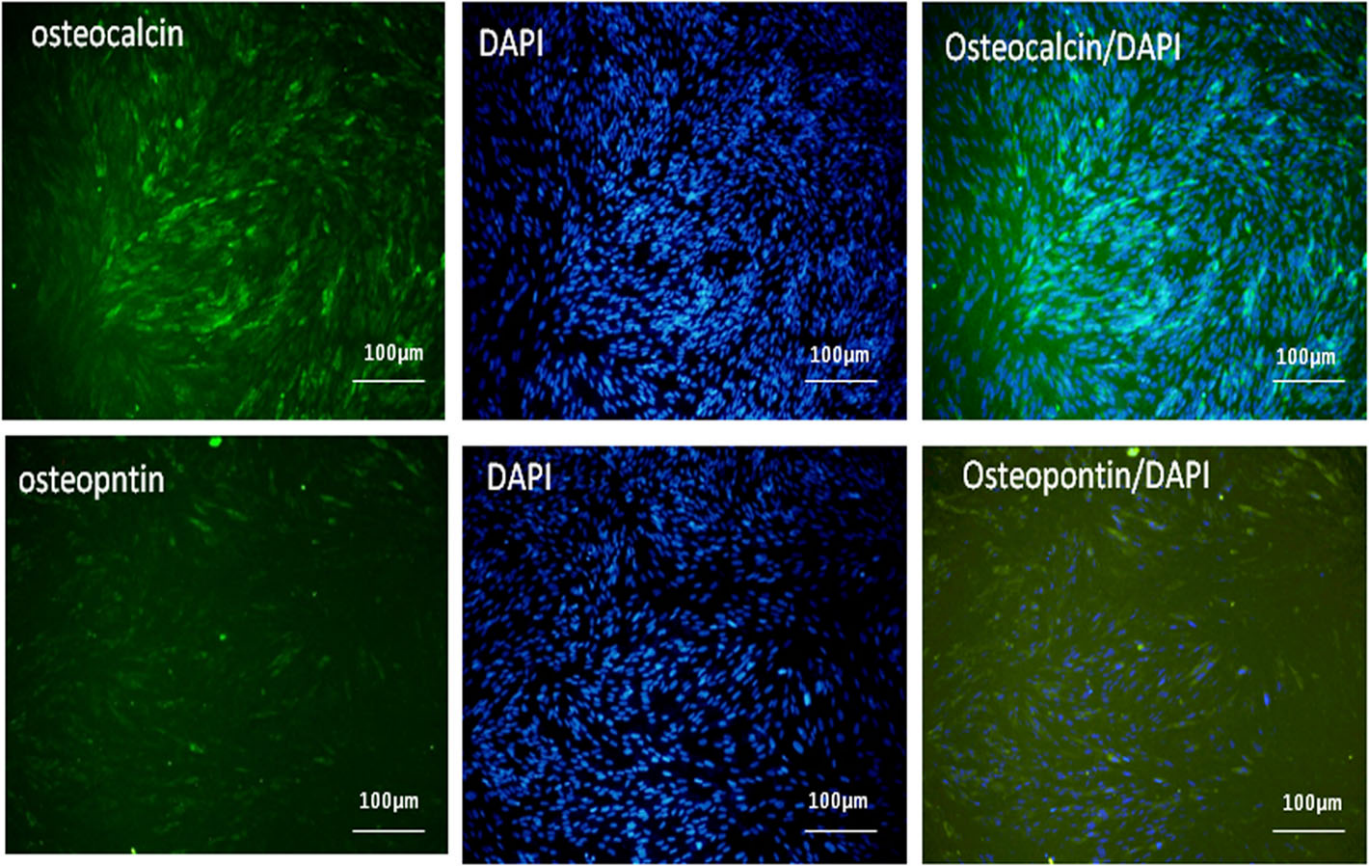
Immunocytochemistry analysis indicated expression of osteocalcin and osteopontin in human endometrial stem cells after being exposed to osteogenic media for 21 days. The nuclei were stained with DAPI

Immunocytochemistry assay was performed on the osteoblast cells differentiated from endometrial stem cells cultured in osteogenic media for 21 days using specific antibodies targeting osteoblast cell markers, such as osteopontin and osteocalcin (Fig. 2). Protein expression of osteopontin and osteocalcin was positive in the treatment group while no positive signal was detected in the control group.

### Alkaline phosphatase activity

**Fig. 3:**
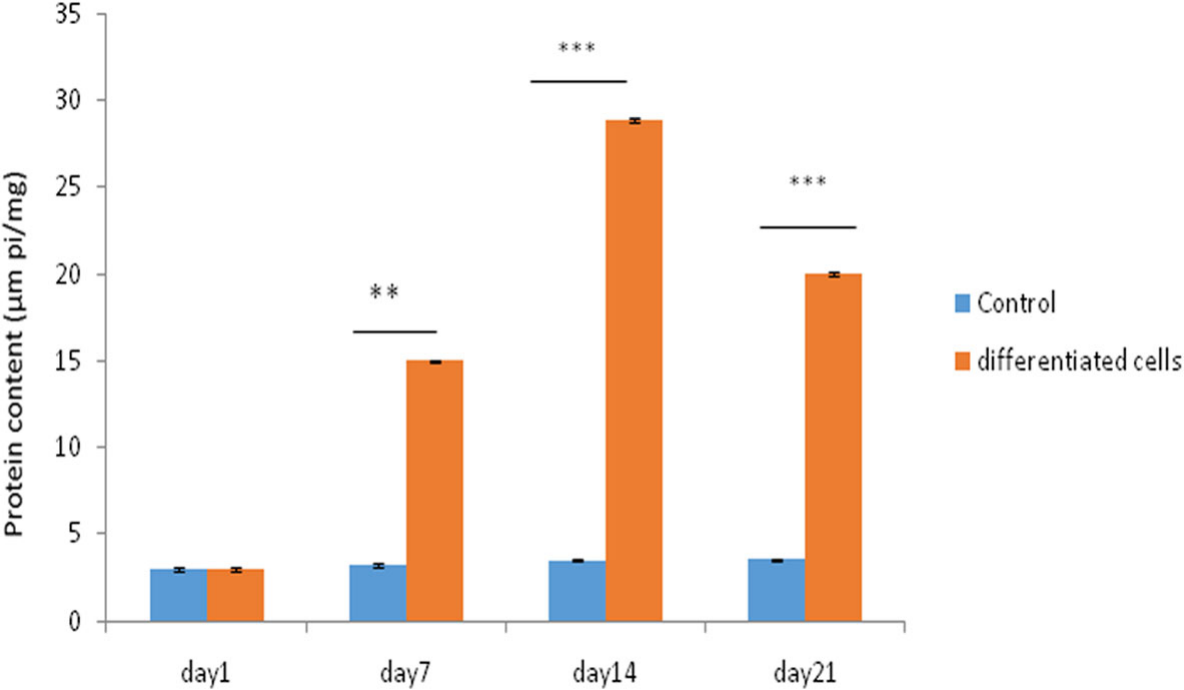
Alkaline phosphatase production of EnSCs. Control—tissue culture polystyrene (TPS). Expression of alkaline phosphatase in differentiation group was higher than that in ESCs as the control group. Expression of alkaline phosphatase reached its peak on day 14 while it was reduced on day 21. All data are expressed as a mean of three experiments ± 1 standard deviation. (*p < 0.05; **p < 0.01; ***p < 0.001)

**Fig. 4:**
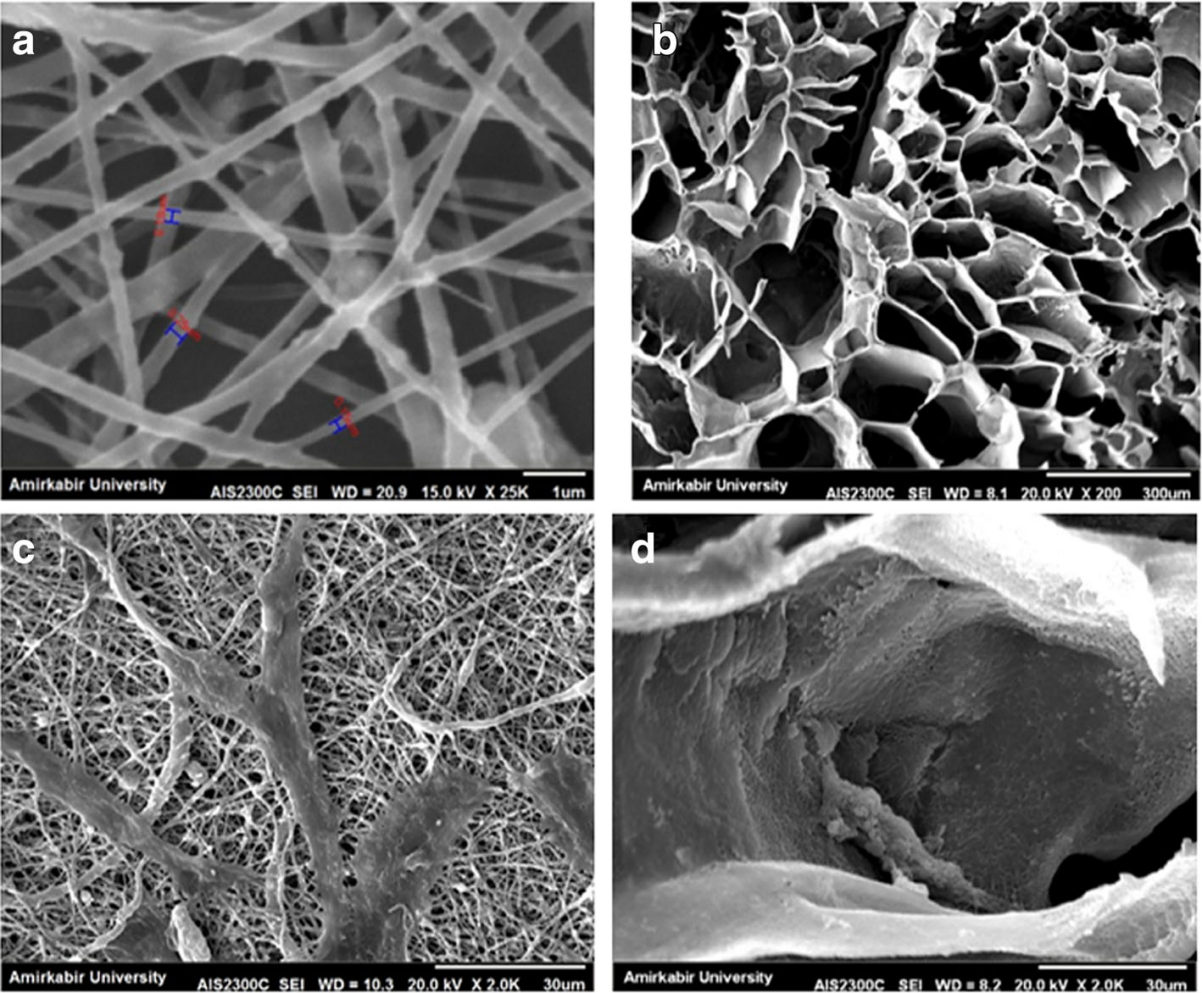
SEM images (**a** [1μm], **b** [300μm]) obtained from the surface of the synthesized scaffolds, **c**: cell attachment (30μm), **d** (30μm)

The results of the ALP assay are presented in Fig. 3. As shown in Fig. 4, ALP activity of osteoblast cells, derived from human endometrial stem cells, which were cultured on a plate, was higher compared to the control groups. Furthermore, ALP was highest on day 21 compared to the other days.

### Scaffold characterization

#### Scanning electron microscopy

**Fig. 5:**
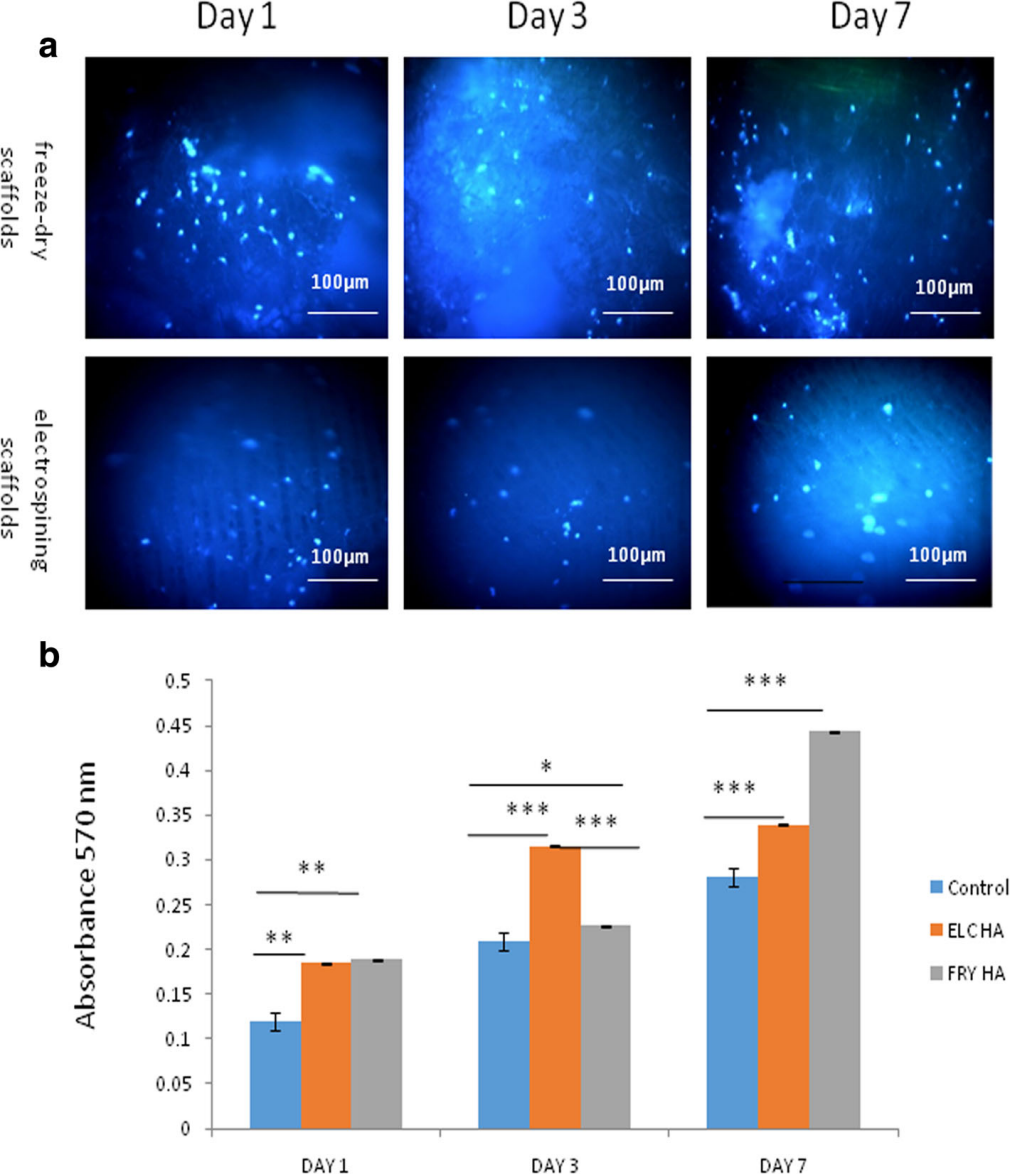
SEM micrographs (**b**, **c**) obtained from endometrial stem cells seeded onto the nanocomposite scaffolds showing adhered cells on the surface of the scaffolds. **a** Fluorescent microscopic results of hEnSCs density on PLGA/HA scaffolds on days 1, 3, and 7. Cells were stained with DAPI. **b** Cell viability measured by MTT assay. All data are expressed as a mean of three experiments ± 1 standard deviation. (*p < 0.05; **p < 0.01; ***p < 0.001)

SEM was performed to evaluate the morphology of the nanocomposites. The SEM results which were used to study the surfaces of the prepared porous nanocomposite scaffolds are shown in Fig. 4. These results demonstrated that electrospinning of PLGA/HA nanofibers was beadless and smooth, and no branching was observed (Fig. 5a). The average diameter of the fibers was 200–800 nm that leads to direct osteogenesis (Fig. 4a). In freeze-dried scaffold, a network of interconnected pores with a uniform honeycomb-like shape was observed. Moreover, the average diameter of the fibers in these scaffolds ranged from 170 to 370 nm, which is optimal for bone cell growth. Furthermore, based on the SEM micrograph of PLGA/HA nanofibers, the structure of the freeze-dried scaffolds was more porous compared to the electrospun scaffolds. Therefore, freeze-drying is possibly a better approach for scaffold fabrication (Fig. 4b).

### Cell adhesion and proliferation on the scaffolds

The SEM results of cell culture onto the nanocomposite scaffold samples for 3 days are presented in Fig. 5. Electronic microscopy micrographs have demonstrated that cell adhesion, growth, and spread occurred both on the freeze-dried and electrospun PLG/HA scaffolds. The cells cultured on the electrospun PLGA/HA nanofiber aligned along the main axis of the fibers (Fig. 4c) while the cells cultured on the freeze-dried PLGA/HA scaffolds completely penetrated into the pores (Fig. 4d). In addition, SEM images illustrated the excellent adhesion and integration of the cultured cells on both scaffolds. These results showed that PLGA/HA could potentially be an appropriate scaffold used for differentiation of hEnSCs into osteoblast-like cells.

### Cell viability and survival assay

DAPI staining of the cultured cells onto the PLGA/HA scaffolds on days 1, 3, and 7 of culture demonstrated that cells were attached onto both of the scaffolds (Fig. 5a). MTT assay was performed to investigate the viability of hEnSCs in connection with the mentioned scaffolds on days 1, 3, and 7 of culture. The results showed that none of the scaffolds had any negative effects on the proliferation rate of the cultured cells compared to the plastic surfaces (Fig. 5b). Furthermore, the fabricated scaffolds could preserve the biocompatibility of the scaffolds, and the viability of the derived cells was increased gradually in 3D culture compared to the 2D culture. However, cell viability of the cells cultured onto the freeze-dried scaffolds was considerably higher than that of the electrospun scaffolds (*p* < 0.001).

### Real-time PCR

**Fig. 6:**
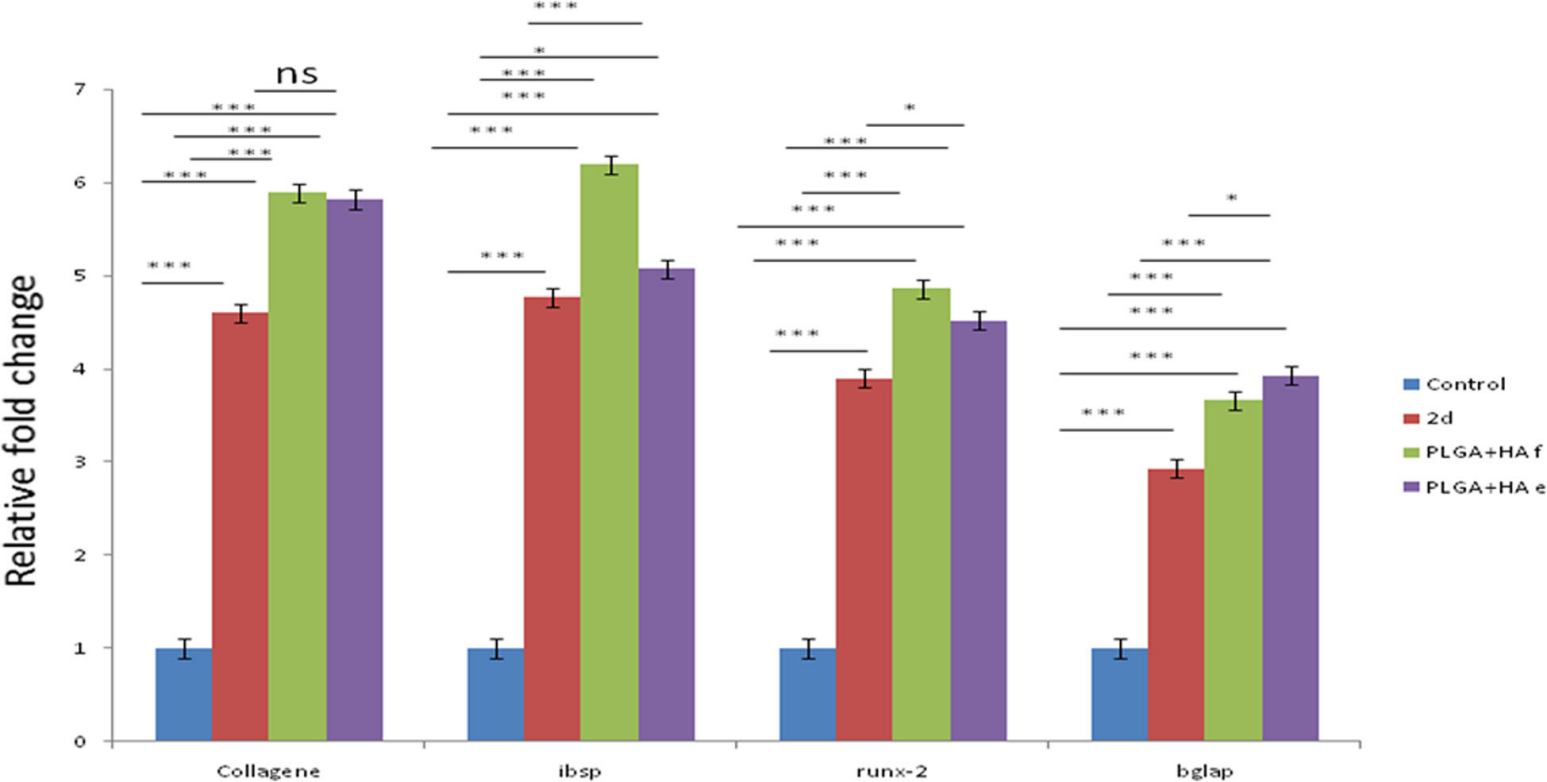
Quantitative mRNA expression analysis of osteoblast-like cells derived from hEnSCs seeded onto PLGA/HA scaffolds after 21 days. Results showed that the expression of osteoblast markers in the differentiated cells onto the freeze-dried PLGA/HA scaffold was higher than those in the cells cultured onto the electrospun scaffold, specially IBSP (*p* < 0.001) and RUNX2 (*p* < 0.001). The expression of BGLAP in the cells cultured onto the electrospun scaffold was higher than that of detected in cells cultured onto the freeze-dried scaffold (*p* < 0.001). All data are expressed as a mean of three experiments ± 1 standard deviation. (*p < 0.05; **p < 0.01; ***p < 0.001)

Real-time PCR was performed to investigate the expression of osteoblast-specific markers at mRNA level after 21 days of culture. It was also performed on day 7 of seeding the cells onto the PLGA/HA scaffolds. Gene expression was examined in cells cultured both in the 2D and 3D culture media. Genes that were anticipated to be express during differentiation included collagene type 1, IBSP, Runx2, and BGLAP. The results of real-time PCR showed that osteoblast cells, derived from human endometrial stem cells, expressed the phenotypic markers of the osteoblast cells after being treated with the osteogenic culture media. As shown in Fig. 6, cells in 3D medium expressed markers more than those cultured in 2D medium (*p* < 0.001). Furthermore, it has been found that expression levels of IBSP and Runx2, as an osteoblast precursor cell markers, were higher in cells cultured onto the freeze-dried scaffold compared to those cultured onto the electrospun scaffold (Fig. 6). Higher expression levels of the osteoblast-specific markers in cells cultured on to the freeze-dried scaffold illustrated that these scaffolds are more capable to maintain the osteoblast-like cells derived from hEnSCs compared to the electrospun scaffolds. Therefore, freeze-dried PLGA/HA scaffolds provide a more suitable topographic situation for osteoblast differentiation.

## Discussion

The first purpose of this study was to differentiate hEnSCs to osteoblast cells using an osteogenic medium which was evaluated using ALP secretion, calcium deposition, and ICC. Secondly, various behavioral aspects of differentiated human osteoblast cells on the surface of both electrospun and freeze-dried PLGA/HA nanocomposite scaffolds were evaluated and compared in terms of gene expression, cell proliferation, attachment, and morphology. In this study, the evaluation of osteoblast gene expression showed that PLGA/HA scaffold promotes differentiation of hEnSCs into osteoblast cells, more effectively, compared to the 2D cell culture environment. PLGA is one of the most significantly developed biodegradable synthetic polymers which has been approved by the FDA and is widely used [37]. Furthermore, HA has been proven to be both osteoinductive and osteoconductive which make it a suitable choice for bone replacement scaffolds due to its chemical similarities to the inorganic materials found in the bone tissue [14]. Scaffolds can provide a three-dimensional structure for ingrowths of the cells and act as a temporary component for extracellular matrix and should have both the appropriate structural and functional properties [38–40].

Differentiation of hEnSCs into the osteoblast-like cells was certified in terms of morphological and molecular criteria. The findings of the present study concerning osteoblast-specific gene marker expression, alizarin red, and ALP demonstrated that hEnSCs have the potential to differentiate into the osteoblast-like cells. The results of IHC investigation demonstrated that osteopontin and osteocalcin were expressed in the cells treated with osteogenic medium. Mineralization detected in this study was in accordance with those reported in the previous studies on hEnSCs, suggesting same as osteogenic potentials [41]. According to a previously published report, PLGA/HA 3D composite scaffolds demonstrated better performance with respect to mineral deposition and osteogenesis either cultured with osteoblasts in vitro [42]. Therefore, PLGA nanofibers integrated with the nano-HA are more suitable to be used as a biomimetic scaffold for bone tissue regeneration. Moreover, it has been demonstrated that the PLGA/HA scaffold enhanced osteoblastic cell growth, proliferated and differentiation as well as Hydroxyapatite [43, 44]. In addition, the data obtained in the present study confirm the findings of the mentioned reports, and it has been demonstrated that HA that excited in both electrospun and freeze-dried PLGA/HA nanocomposite scaffolds influenced the differentiation of hEnSCs into osteoblast which was confirmed by real-time PCR. According to a previously published report, the integration of the HA with the PLGA nanofibers using electrospinning method is a notable way to obtain nanofibrous scaffolds with more appropriate biological and physical performances, which are critical for bone regeneration [45].

Obtained data from cytotoxicity tests or cell viability assays confirmed the ability of both electrospun and freeze-dried PLGA/HA nanocomposites to support cell viability. The whole set of evaluated nanocomposites displayed comparable biocompatibility, and the cell proliferation rates of osteoblast cells in various groups were higher than that of the control group; however, cell viability on freeze-dried PLGA/HA scaffold was higher compared to that on the electrospun PLGA/HA scaffold, on day 7 of being seeded (*p* < 0.001). Based on SEM results, it can be mentioned that the hEnSCs seeded onto both nanocomposites presented good adhesion and proliferation as well as spreading morphology which was regular for these cells. Moreover, spreading and adhesion of hEnSCs, which were verified to occur on both of the nanocomposites examined in the present study, can be dependent on the approved biocompatibility and non-cytotoxicity of both samples. Fiber diameter and pore size of the scaffold are another characteristics that play an important role in cell adhesion and differentiation [46, 47]. The fiber diameter of PLGA in the present study was nearly 25–30 μm. The size of osteoblasts is nearly 10–30 μm and cell proliferation takes place in average pore size of 50 μm, while cells can migrate extensively in larger pores up to 100 μm. An average pore size of greater than 300 μm has been recommended to be used due to the enhanced bone regeneration and the formation of capillaries [48]. Consequently, both the electrospun and freeze-dried PLGA/HA scaffolds provide appropriate physiological in vitro nanoenvironment for the differentiation of hEnSCs. However, it should be noted that cell attachment and growth were better on the freeze-dried scaffold compared to those on the elctrospun scaffold. SEM showed that the freeze-dried PLGA/HA scaffolds have porous structure versus the fibrous structure of the electrospun PLGA/HA scaffolds. Freeze-dried scaffolds showed better cell permeation, and their larger pore size, higher porosity, and interconnection result in an increased cell proliferation rate. However, the compressive mechanical property of the freeze-dried scaffolds, which make it acceptable to be used for bone replacement, was lower than that of the other scaffolds fabricated using different approaches [49]. Thus, the cell growth and proliferation on the freeze-dried scaffolds provide a more suitable microenvironment for cells during proliferation compared to electrospun nanofibrous. Finally, it is demonstrated that freeze-dried PLGA/HA nanocomposites are significantly biocompatible without exerting any notable cytotoxic effects and influence the differentiation of hEnSCs into osteoblast-like cell more compared to the electrospun scaffold; therefore, they could be used as potential biomaterials for bone tissue engineering applications.

The results of ALP activity assay and evaluation of expression of osteoblast-specific cell markers at mRNA and protein levels showed that both scaffolds promote differentiation of hEnSCs into osteoblast-like cells. Therefore, both scaffolds may be potential candidates to be applied for the treatment of bone disorders using bone tissue engineering. However, differentiation of hEnSCs into osteoblast-like cell onto the freeze-dried scaffold was better than that onto the electrospun scaffold (*p* < 0.001).

This study has some limitations. PLGA is widely used in bone tissue engineering due to its biodegradability rate and appropriate physicochemical properties. However, mineralization is difficulty occurred by the synthetic polymers such as PLGA. This limitation results from the inadequate ionic molecular group in synthetic polymers.

## Conclusion

In the present study, human endometrial stem cells could successfully be differentiated into osteoblast-like cells using the osteogenic medium. This may propose that hEnSCs are an attractive alternative for the repair of bone tissue defects, as they display several significant and potential advantages over other stem cells. Moreover, nanocomposite scaffolds were successfully fabricated using electrospinning and freeze-drying methods. Furthermore, it has been shown that both the freeze-dried and electrospun PLGA/HA nanocomposite scaffolds have appropriate properties to support the attachment and proliferation of differentiated osteoblast cells. Cells that were cultured onto the freeze-dried PLGA/HA scaffolds showed significantly higher cell viability compared to those cultured onto the electrospun PLGA/HA scaffolds. Moreover, the integration of the HA with the PLGA to fabricate electrospun and freeze-dried scaffolds is a considerable way to prepare nanocomposite scaffolds with better physical and biological performances, which are more appropriate for bone regeneration. Therefore, the results of the present study highlight the potential application of these nanocomposite scaffolds in bone tissue repair processes.

## Abbreviations

ARS: Alizarin red stain
DPBS: Dulbecco’s phosphate
ES: Embryonic stem cells
FDA: Food and Drug Administration
hEnSCs: Human endometrial stem cells
HSC: Hematopoietic stem cells
ICC: Immunocytochemistry
IHC: Immunohistochemistry
MSCs: Mesenchymal stem cells
MTT: 3-(4,5-Dimethylthiazoyl-2-yl)-2,5-diphenyltetrazolium bromide
PLGA: Poly(lactic-co-glycolic acid)
PLGA/HA: Polylactide-co-glycolide/hydroxyapatite
SEM: Scanning electron microscopy
